# Chimeric Anti-Glypican 1 Antibodies Exert Antitumor Activities in Xenograft Models of Lung and Pancreatic Cancers

**DOI:** 10.3390/ijms27104181

**Published:** 2026-05-08

**Authors:** Haruto Yamamoto, Hiroyuki Suzuki, Tomokazu Ohishi, Hiroyuki Satofuka, Mika K. Kaneko, Yukinari Kato

**Affiliations:** 1 Department of Antibody Drug Development, Tohoku University Graduate School of Medicine, 2-1 Seiryo-machi, Aoba-ku, Sendai 980-8575, Japan; yamamoto.haruto.s4@dc.tohoku.ac.jp (H.Y.); hiroyuki.satofuka.e1@tohoku.ac.jp (H.S.); mika.kaneko.d4@tohoku.ac.jp (M.K.K.); 2 Institute of Microbial Chemistry (BIKAKEN), Laboratory of Oncology, Microbial Chemistry Research Foundation, 3-14-23 Kamiosaki, Shinagawa-ku, Tokyo 141-0021, Japan; ohishit@bikaken.or.jp

**Keywords:** Glypican-1, monoclonal antibody therapy, ADCC, CDC, lung cancer, pancreatic cancer

## Abstract

Glypican-1 (GPC1) has emerged as a critical mediator of malignant tumor progression. GPC1 plays essential roles in regulating various signaling pathways involved in tumor cell proliferation, invasiveness, and tumorigenesis. Overexpression of GPC1 in tumors mediates oncogenic transformation, epithelial-to-mesenchymal transition, metastatic dissemination, and therapeutic resistance. Accordingly, GPC1-targeted therapeutic strategies have been investigated in clinical and preclinical studies. However, clinical efficacy has been limited. We previously developed an anti-GPC1 monoclonal antibody (mAb), G_1_Mab-28 (mouse IgG_1_, κ), which exhibits high affinity and specificity for GPC1. In the present study, we generated recombinant isotype-converted G_1_Mab-28, including G_1_Mab-28-mG_2a_ (mouse IgG_2a_) and G_1_Mab-28-hG_1_ (human IgG_1_). Both mAbs recognized GPC1-expressing human tumor cell lines, including lung squamous cell carcinoma PC-10 and pancreatic ductal adenocarcinoma PK-45H, by flow cytometry. Moreover, both mAbs exerted antibody-dependent cellular cytotoxicity and complement-dependent cytotoxicity against those cell lines. In mouse xenograft models, treatment with the mAbs resulted in potent antitumor efficacy against PC-10 and PK-45H tumors. Collectively, these findings support the therapeutic potential of G_1_Mab-28 for the treatment of GPC1-positive tumors.

## 1. Introduction

Glypican-1 (GPC1) is an extracellular matrix-associated heparan sulfate proteoglycan and serves as a co-receptor for fibroblast growth factors, hepatocyte growth factor, some Wnt ligands, and TGF-β to enhance the signaling pathways [1,2,3,4,5,6]. GPC1 plays essential roles in tumor cell proliferation, invasiveness, epithelial-to-mesenchymal transition, stemness, and therapeutic resistance [3,7,8]. The overexpression of GPC1 is significantly associated with reduced overall survival, disease-free survival, and/or relapse-free survival in esophageal squamous cell carcinoma [9]. Furthermore, the GPC1 overexpression has been reported in gliomas, lung squamous cell carcinoma (LSCC), breast cancer, prostate cancer, and pancreatic ductal adenocarcinoma (PDAC). In these tumors, a strong correlation between GPC1 overexpression and poor clinical outcomes has been reported [10,11,12,13,14].

In research and development for a therapeutic antibody, costs range from $1 billion to over $2 billion per approved product [15]. Several anti-GPC1 monoclonal antibodies (mAbs) have been developed in both preclinical and clinical studies [7,16,17,18]. A chimeric antibody, Miltuximab, was developed from an anti-GPC1 mAb (clone MIL-38) [17], which was generated by immunization with the UCRU-BL-17CL, a human bladder cancer cell line [19]. The first-in-human clinical trial of Miltuximab demonstrated its safety and tolerability in patients with advanced PDAC, bladder cancer, and prostate cancer (ACTRN12616000787482) [20]. However, the clinical development of Miltuximab was discontinued. Miltuximab has been further developed as an immunotheranostic agent ([^67^Ga]Ga-DOTA-Miltuximab), and its safety and tolerability have been evaluated in patients with advanced solid tumors [21]. In addition, ^89^Zr-DFO-Miltuximab has been established as an effective immuno-positron emission tomography imaging probe for the detection of GPC1-positive glioblastoma in mouse models [22]. Radiolabeled Miltuximab, including ^225^Ac- and ^177^Lu-labeled forms, has been developed for α- and β-emitting radionuclide therapies, respectively [17,23]. Moreover, a photoimmunotherapy agent, Miltuximab-IR700, has shown a significant reduction in the viability of GPC1-positive cancer cell lines [24].

Beyond these formats, a bispecific T-cell engager, MIL-38-CD3 BiTE, was engineered in a tandem single-chain variable fragment (scFv) configuration by linking the scFv of Miltuximab to an anti-CD3 scFv. This construct effectively redirected T-cell-mediated cytotoxicity toward GPC1-expressing prostate cancer cells in preclinical models [25].

An anti-GPC1 mAb (clone 1–12) exhibited the antitumor efficacy of an esophageal cancer preclinical model via the antibody-dependent cellular cytotoxicity (ADCC) and complement-dependent cytotoxicity (CDC) activity [26]. Furthermore, another anti-GPC1 mAb (clone 01a033) was generated and exhibited a high internalizing activity suitable for antibody–drug conjugates (ADCs) [27,28]. A humanized version of 01a033 (clone T2) was also developed to ADC, which have been evaluated and demonstrated antitumor efficacy in mouse models of gastric cancer, esophageal cancer, glioblastoma, and PDAC [29,30,31]. Additionally, a dromedary camel VHH nanobody (D4)-based chimeric antigen receptor (CAR) T-cells targeting GPC1 has shown promising antitumor activity in mouse models of PDAC [32].

To target GPC1, our group has developed mAbs against GPC1 (G_1_Mabs) using flow cytometry-based high-throughput screening. Among 124 clones of G_1_Mabs, a clone G_1_Mab-28 (mouse IgG_1_, κ) specifically recognized GPC1, but not other GPCs in flow cytometry [33]. Therefore, G_1_Mab-28 possesses potential for tumor therapy. In this study, we isotype-converted G_1_Mab-28 into G_1_Mab-28-mG_2a_ (mouse IgG_2a_-type) and G_1_Mab-28-hG_1_ (human IgG_1_-type) and evaluated the ADCC, CDC, and in vivo antitumor efficacy against GPC1-positive tumors.

## 2. Results

### 2.1. Production of Isotype-Converted mAbs from G_1_Mab-28

**Figure 1:**
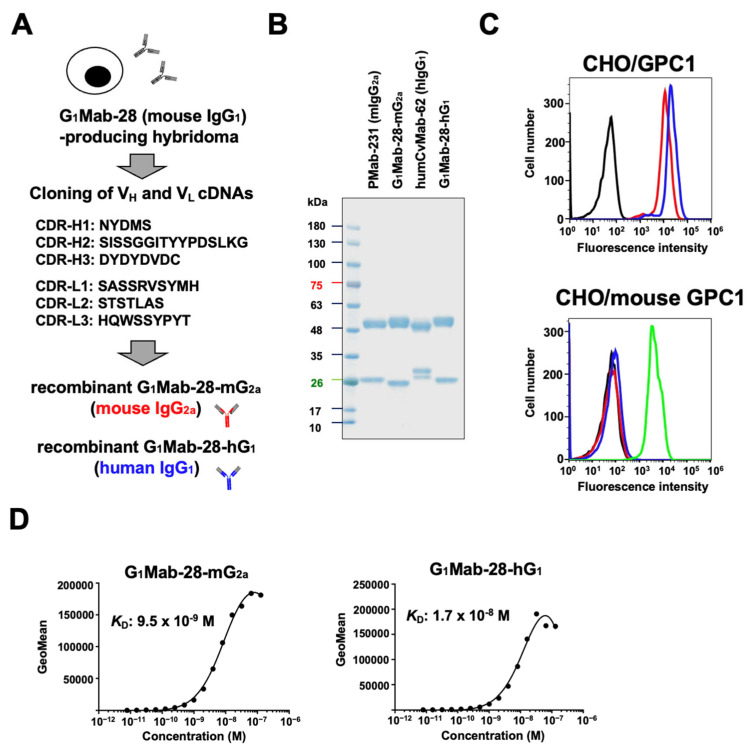
Production of recombinant G_1_Mab-28-mG_2a_ and G_1_Mab-28-hG_1_. (**A**) After determination of CDRs of G_1_Mab-28 (mouse IgG_1_), G_1_Mab-28-mG_2a_ (mouse IgG_2a_) and G_1_Mab-28-hG_1_ (human IgG_1_) were produced. The amino acid sequence of V_H_ and V_L_ CDRs was indicated. (**B**) PMab-231 (control mIgG_2a_), G_1_Mab-28-mG_2a_, humCvMab-62 (control hIgG_1_), and G_1_Mab-28-hG_1_ were subjected to SDS-PAGE, and the gel was stained with Bio-Safe CBB G-250 Stain. (**C**) Flow cytometry using G_1_Mab-28-mG_2a_ (1 μg/mL; Red line) and G_1_Mab-28-hG_1_ (1 μg/mL; Blue line) against CHO/GPC1 and CHO/PA16-mouse GPC1 (CHO/mouse GPC1). An anti-PA16 tag mAb (NZ-1) detected PA16-tagged mouse GPC1 (1 μg/mL; Green line). After treatment with primary mAbs or buffer control (Black line), cells were treated with Alexa Fluor 488-conjugated anti-mouse or rat IgG, or FITC-conjugated anti-human IgG. (**D**) Determination of the binding affinity of G_1_Mab-28-mG_2a_ and G_1_Mab-28-hG_1_. CHO/GPC1 was suspended in G_1_Mab-28-mG_2a_ and G_1_Mab-28-hG_1_ at indicated concentrations, followed by Alexa Fluor 488-conjugated anti-mouse IgG or FITC-conjugated anti-human IgG treatment. The SA3800 Cell Analyzer was used to analyze fluorescence data. The dissociation constant (*K*_D_) values were determined using GraphPad Prism 6.

We previously reported that G_1_Mab-28, an anti-GPC1 mAb, detects GPC1-positive cells by flow cytometry, Western blotting, and immunohistochemistry. Furthermore, G_1_Mab-28 did not show cross-reactivity to other five glypicans (GPC2 to GPC6) in flow cytometry [33]. We next cloned the cDNA of G_1_Mab-28 and determined the CDR sequences (Figure 1A). Subsequently, a mouse IgG_2a_-type G_1_Mab-28 (G_1_Mab-28-mG_2a_) and human IgG_1_-type G_1_Mab-28 (G_1_Mab-28-hG_1_) were generated by fusing the V_H_ and V_L_ of G_1_Mab-28 with the C_H_ and C_L_ chains of mouse IgG_2a_ and human IgG_1_, respectively (Figure 1A). A mouse IgG_2a_ isotype control mAb, PMab-231 (referred to as control mIgG_2a_) and a human IgG_1_ isotype control mAb, humCvMab-62 (referred to as control hIgG_1_) were also produced. The purity of original and recombinant mAbs was confirmed by SDS-PAGE under reduced conditions (Figure 1B). We also confirmed that G_1_Mab-28-mG_2a_ and G_1_Mab-28-hG_1_ reacted with human GPC1-overexpressed Chinese hamster ovary-K1 (CHO/GPC1), but did not cross-react with mouse GPC1-overexpressed CHO-K1 (89% sequence identity to human GPC1 [26], Figure 1C) nor CHO-K1 (Supplementary Figure S1) in flow cytometry.

Next, the binding affinity of G_1_Mab-28-mG_2a_ and G_1_Mab-28-hG_1_ was determined using flow cytometry. The dissociation constant (*K*_D_) values of G_1_Mab-28-mG_2a_ and G_1_Mab-28-hG_1_ for CHO/GPC1 were determined to be 9.5 × 10^−9^ M and 1.7 × 10^−8^ M, respectively (Figure 1D). These results indicated that G_1_Mab-28-mG_2a_ and G_1_Mab-28-hG_1_ possess higher binding affinity compared to parental mAb, G_1_Mab-28 as reported previously (*K*_D_: 3.3 × 10^−8^ M) [33].

### 2.2. Flow Cytometry Using G_1_Mab-28-mG_2a_ and G_1_Mab-28-hG_1_ in GPC1-Positive Cancer Cells

**Figure 2:**
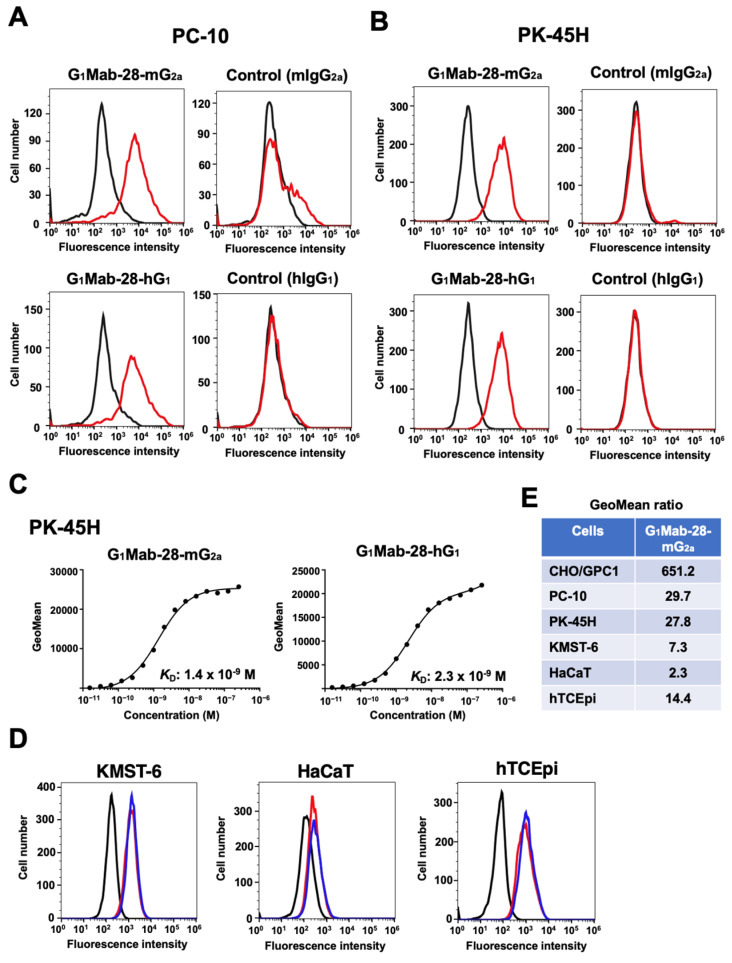
Reactivity of G_1_Mab-28-mG_2a_ and G_1_Mab-28-hG_1_ to tumor cells. (**A**,**B**) Flow cytometry using control mIgG_2a_, G_1_Mab-28-mG_2a_, control hIgG_1_, and G_1_Mab-28-hG_1_ (1 μg/mL; Red line) or buffer control (Black line) against LSCC PC-10 (**A**) and PDAC PK-45H (**B**). After treatment with primary mAbs, cells were treated with Alexa Fluor 488-conjugated anti-mouse IgG or FITC-conjugated anti-human IgG. (**C**) Determination of the binding affinity of G_1_Mab-28-mG_2a_ and G_1_Mab-28-hG_1_. PK-45H was suspended in G_1_Mab-28-mG_2a_ and G_1_Mab-28-hG_1_ at indicated concentrations, followed by Alexa Fluor 488-conjugated anti-mouse IgG or FITC-conjugated anti-human IgG treatment. Fluorescence data were analyzed using the SA3800 Cell Analyzer. The *K*_D_ values were determined using GraphPad Prism 6. (**D**) Flow cytometry using control G_1_Mab-28-mG_2a_ (1 μg/mL; Red line), G_1_Mab-28-hG_1_ (1 μg/mL; Blue line), or buffer control (Black line) against HaCaT, KMST-6, and hTCEpi. After treatment with primary mAbs, cells were treated with Alexa Fluor 488-conjugated anti-mouse IgG or FITC-conjugated anti-human IgG. (**E**) The GeoMean (G_1_Mab-28-mG_2a_) ratio to buffer control was quantified.

We previously screened the GPC1-positive tumor cell lines using flow cytometry. Among them, we chose human LSCC cell lines such as PC-10 and PDAC PK-45H based on their expression of GPC1 and their availability in mouse xenograft models. As shown in Figure 2A,B, G_1_Mab-28-mG_2a_ reacted with PC-10 and PK-45H at 1 µg/mL. In contrast, control mIgG_2a_ did not. G_1_Mab-28-hG_1_ also showed similar reactivity at 1 µg/mL, but control hIgG_1_ did not (Figure 2A,B). The K_D_ values of G_1_Mab-28-mG_2a_ and G_1_Mab-28-hG_1_ for PK-45H were determined to be 1.4 × 10^−9^ M and 2.3 × 10^−9^ M, respectively (Figure 2C), indicating that both G_1_Mab-28-mG_2a_ and G_1_Mab-28-hG_1_ exhibit moderate binding affinity to PK-45H.

We next examined GPC1 expression in non-tumor cells. As shown in Figure 2D, G_1_Mab-28-mG_2a_ and G_1_Mab-28-hG_1_ reacted to fibroblast KMST-6, keratinocyte HaCaT, and corneal epithelial hTCEpi. The GeoMean (G_1_Mab-28-mG_2a_) ratio to buffer control was quantified in Figure 2E. Tumor cell lines (PC-10 and PK-45H) express higher level of GPC1 compared to non-tumor cell lines (KMST-6, HaCaT, and hTCEpi).

### 2.3. G_1_Mab-28-mG_2a_ Elicited ADCC and CDC Against GPC1-Positive Cells

**Figure 3:**
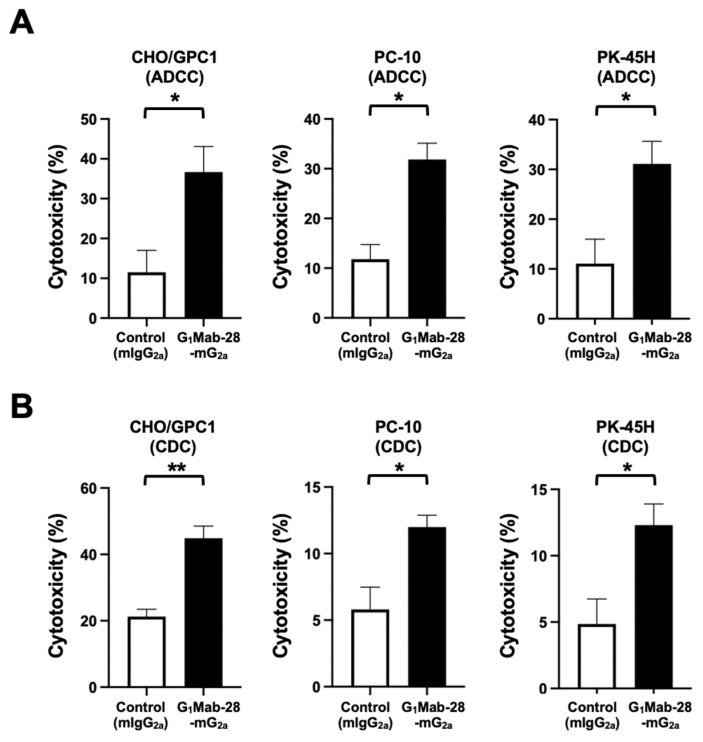
ADCC and CDC by G_1_Mab-28-mG_2a_ against GPC1-positive tumor cells. The target cells labeled with Calcein AM (CHO/GPC1, PC-10, and PK-45H) were incubated with effector splenocyte derived from BALB/c nude mice (**A**) or rabbit complement (**B**) in the presence of G_1_Mab-28-mG_2a_ or control mIgG_2a_. Calcein release into the medium was measured, and cytotoxicity was determined. Values are shown as the mean ± SEM. Asterisks indicate statistical significance (* *p* < 0.05, ** *p* < 0.01; two-tailed unpaired *t*-test).

ADCC and CDC induced by G_1_Mab-28-mG_2a_ against GPC1-positive CHO/GPC1, PC-10, and PK-45H cells were investigated. The ADCC induced by G_1_Mab-28-mG_2a_ was evaluated in the presence of effector splenocytes derived from BALB/c nude mice compared with control mIgG_2a_. As shown in Figure 3A, G_1_Mab-28-mG_2a_ elicited potent ADCC against CHO/GPC1 (36.7% cytotoxicity; *p* < 0.05) compared with the control mIgG_2a_ (11.5% cytotoxicity). G_1_Mab-28-mG_2a_ induced ADCC against PC-10 (31.8% cytotoxicity; *p* < 0.05) more effectively than the control mIgG_2a_ (11.8% cytotoxicity). Furthermore, G_1_Mab-28-mG_2a_ also induced ADCC against PK-45H (31.1% cytotoxicity; *p* < 0.05) more effectively than the control mIgG_2a_ (11.1% cytotoxicity).

The CDC elicited by G_1_Mab-28-mG_2a_ was next evaluated. Rabbit complement is used for its high activity against human cells inducing membrane attack complex formation and cell lysis [34]. As shown in Figure 3B, G_1_Mab-28-mG_2a_ induced significant CDC against CHO/GPC1 (44.9% cytotoxicity; *p* < 0.01) compared to the control mIgG_2a_ (21.3% cytotoxicity). G_1_Mab-28-mG_2a_ also elicited CDC against PC-10 (12.0% cytotoxicity; *p* < 0.05) more effectively than the control mIgG_2a_ (5.8% cytotoxicity). Additionally, G_1_Mab-28-mG_2a_ showed CDC against PK-45H (12.3% cytotoxicity; *p* < 0.05) more effectively than the control mIgG_2a_ (4.9% cytotoxicity).

We also established GPC1-KO KYSE770 (BINDS-70) (Supplementary Figure S2). However, ADCC and CDC were not elicited by G_1_Mab-28-mG_2a_ (Supplementary Figure S3). These results indicated that G_1_Mab-28-mG_2a_ exerted ADCC and CDC in the presence of effector splenocytes and complements, respectively.

### 2.4. G_1_Mab-28-mG_2a_ Showed Antitumor Effects Against GPC1-Positive Tumor Xenografts

**Figure 4:**
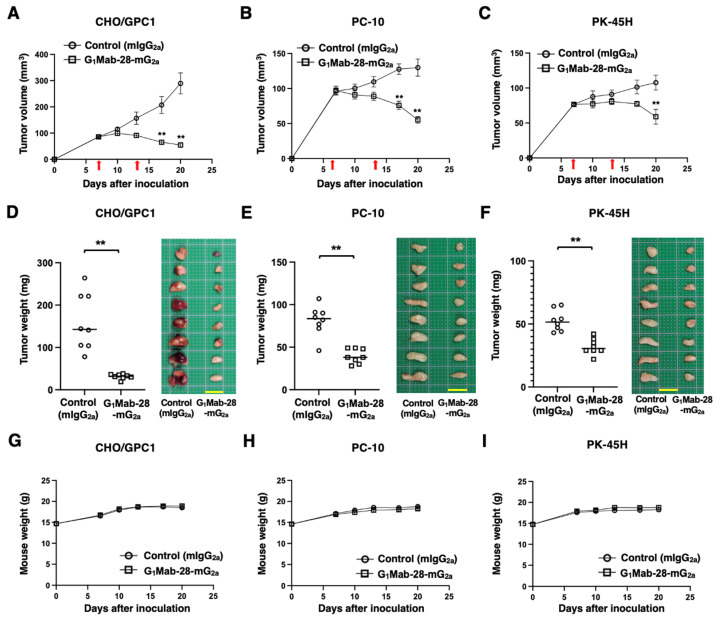
Antitumor activity of G_1_Mab-28-mG_2a_ against human tumor xenografts. (**A**–**C**) CHO/GPC1 (**A**), PC-10 (**B**), and PK-45H (**C**) cells were subcutaneously injected into BALB/c nude mice (day 0). G_1_Mab-28-mG_2a_ (100 μg) or control mIgG_2a_ (100 μg) were intraperitoneally injected into each mouse on days 7 and 13 (arrows). The tumor volume is represented as the mean ± SEM. ** *p* < 0.01 (two-way ANOVA with Sidak’s post hoc test). (**D**–**F**) After cell inoculation, the mice were euthanized on day 20. The tumor weights (left) and appearance (right) of CHO/GPC1 (**D**), PC-10 (**E**), and PK-45H (**F**) xenografts were measured. Values are presented as the mean ± SEM. ** *p* < 0.01 (two-tailed unpaired *t*-test). Scale bar, 1 cm. (**G**–**I**) Body weight (mean ± SEM) of xenograft-bearing mice treated with mAbs is presented. There is no significant difference (two-way ANOVA with Sidak’s post hoc test).

CHO/GPC1, PC-10, or PK-45H were inoculated at the left flanks of BALB/c nude mice (day 0). Subsequently, G_1_Mab-28-mG_2a_ or control mIgG_2a_ was intraperitoneally administered into the tumor-bearing mice on days 7 and 13. The tumor volume was measured on the indicated days. The G_1_Mab-28-mG_2a_ administration resulted in a significant reduction in CHO/GPC1 xenografts on days 17 (*p* < 0.01) and 20 (*p* < 0.01) compared with that of control mIgG_2a_ (Figure 4A). In the PC-10 tumor, a significant reduction was observed on days 17 (*p* < 0.01) and 20 (*p* < 0.01) (Figure 4B). In the PK-45H tumor, a significant reduction was also observed on day 20 (*p* < 0.01) (Figure 4C).

In the tumor weight, G_1_Mab-28-mG_2a_ showed the potent reduction in CHO/GPC1 (80% reduction; *p* < 0.01; Figure 4D), PC-10 (52% reduction; *p* < 0.01; Figure 4E), and PK-45H (39% reduction; *p* < 0.01; Figure 4F) compared with control mIgG_2a_. The resected CHO/GPC1, PC-10, and PK-45H tumors on day 20 are shown in each figure. The tumor-bearing mice did not lose body weight by G_1_Mab-28-mG_2a_ treatment (Figure 4G–I).

### 2.5. G_1_Mab-28-hG_1_ Elicited ADCC and CDC Against GPC1-Positive Cells

**Figure 5:**
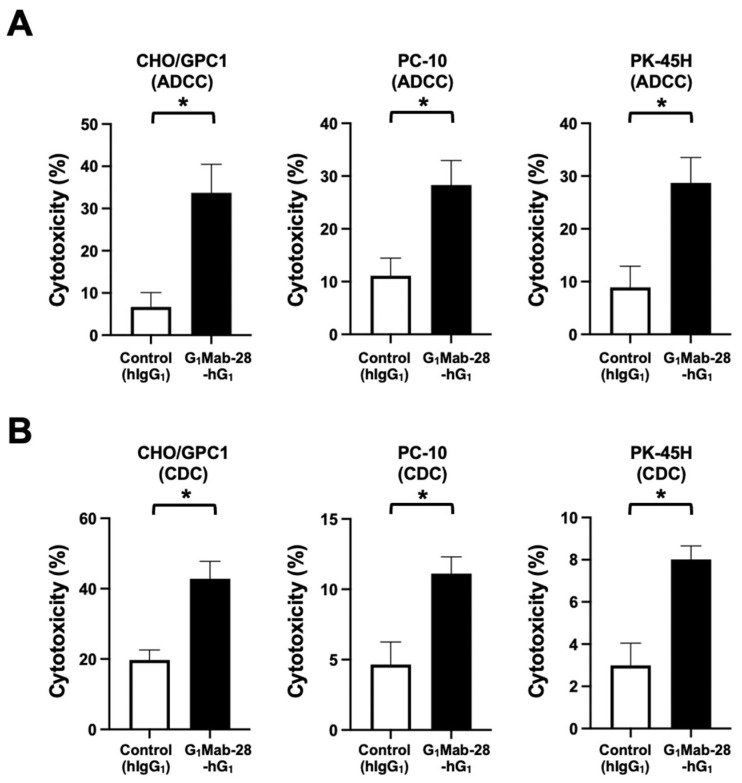
ADCC and CDC by G_1_Mab-28-hG_1_ against GPC1-positive tumor cells. The target cells labeled with Calcein AM (CHO/GPC1, PC-10, and PK-45H) were incubated with effector splenocyte derived from BALB/c nude mice (**A**) or rabbit complement (**B**) in the presence of G_1_Mab-28-hG_1_ or control hIgG_1_. Calcein release into the medium was measured, and cytotoxicity was determined. Values are shown as the mean ± SEM. Asterisks indicate statistical significance (* *p* < 0.05; two-tailed unpaired *t*-test).

ADCC and CDC induced by G_1_Mab-28-hG_1_ against CHO/GPC1, PC-10, and PK-45H cells were next investigated. Since all four mouse Fcγ receptors bind to human IgG_1_ and can elicit ADCC in the presence of mouse effectors [35], the BALB/c nude mice-derived splenocytes were also used as effector cells. The ADCC induced by G_1_Mab-28-hG_1_ and control hIgG_1_ was investigated in the presence of effector splenocytes. As shown in Figure 5A, G_1_Mab-28-hG_1_ induced potent ADCC against CHO/GPC1 (33.7% cytotoxicity; *p* < 0.05) compared to the control hIgG_1_ (6.7% cytotoxicity). G_1_Mab-28-hG_1_ elicited ADCC against PC-10 (28.3% cytotoxicity; *p* < 0.05) more effectively than the control hIgG_1_ (11.1% cytotoxicity). Furthermore, G_1_Mab-28-hG_1_ also showed ADCC against PK-45H (28.7% cytotoxicity; *p* < 0.05) more effectively than the control hIgG_1_ (8.9% cytotoxicity).

The CDC elicited by G_1_Mab-28-hG_1_ and complements was evaluated next. As shown in Figure 5B, G_1_Mab-28-hG_1_ elicited significant CDC against CHO/GPC1 (42.8% cytotoxicity; *p* < 0.05) compared to the control hIgG_1_ (19.7% cytotoxicity). G_1_Mab-28-hG_1_ induced CDC against PC-10 (11.1% cytotoxicity; *p* < 0.05) more effectively than the control hIgG_1_ (4.7% cytotoxicity). Additionally, G_1_Mab-28-hG_1_ showed CDC against PK-45H (8.0% cytotoxicity; *p* < 0.05) more effectively than the control hIgG_1_ (3.0% cytotoxicity).

ADCC and CDC were not elicited by G_1_Mab-28-hG_1_ against BINDS-70 (Supplementary Figure S3). These results indicated that G_1_Mab-28-hG_1_ exerted ADCC and CDC in the presence of effector splenocytes and complements, respectively.

### 2.6. G_1_Mab-28-hG_1_ Showed Antitumor Effects Against GPC1-Positive Tumor Xenografts

**Figure 6:**
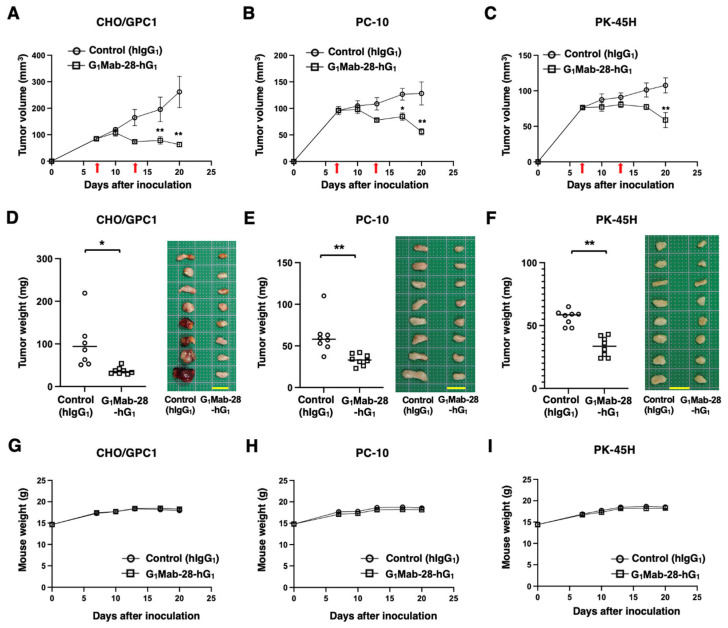
Antitumor activity of G_1_Mab-28-hG_1_ against human tumor xenografts. (**A**–**C**) CHO/GPC1 (**A**), PC-10 (**B**), and PK-45H (**C**) cells were subcutaneously injected into BALB/c nude mice (day 0). G_1_Mab-28-hG_1_ (100 μg) or control hIgG_1_ (100 μg) were intraperitoneally injected into each mouse on days 7 and 13 (arrows). The tumor volume is represented as the mean ± SEM. * *p* < 0.05, ** *p* < 0.01 (two-way ANOVA with Sidak’s post hoc test). (**D**–**F**) After cell inoculation, the mice were euthanized on day 20. The tumor weights (left) and appearance (right) of CHO/GPC1 (**D**), PC-10 (**E**), and PK-45H (**F**) xenografts were measured. Values are presented as the mean ± SEM. * *p* < 0.05, ** *p* < 0.01 (two-tailed unpaired *t*-test). Scale bar, 1 cm. (**G**–**I**) Body weight (mean ± SEM) of xenograft-bearing mice treated with the mAbs is presented. There is no significant difference (two-way ANOVA with Sidak’s post hoc test).

In preclinical studies of trastuzumab (human IgG_1_), a clinically approved anti-HER2 mAb, the antitumor efficacy was evaluated in nude mice in the absence of human-derived effectors [36,37,38]. Therefore, the antitumor effect of G_1_Mab-28-hG_1_ was examined in tumor xenografts inoculated in nude mice. After the inoculation of CHO/GPC1, PC-10, or PK-45H in BALB/c nude mice, G_1_Mab-28-hG_1_ or control hIgG_1_ was intraperitoneally administrated into the tumor-bearing mice on days 7 and 13. The G_1_Mab-28-hG_1_ administration resulted in a reduction in CHO/GPC1 xenografts on days 17 (*p* < 0.01) and 20 (*p* < 0.01) compared with that of control hIgG_1_ (Figure 6A). In the PC-10 tumor, a significant reduction was observed on days 17 (*p* < 0.05) and 20 (*p* < 0.01) (Figure 6B). In the PK-45H tumor, a significant reduction was also observed on day 20 (*p* < 0.01) (Figure 6C).

In the tumor weight, G_1_Mab-28-hG_1_ showed the reduction in CHO/GPC1 (72% reduction; *p* < 0.05; Figure 6D), PC-10 (46% reduction; *p* < 0.01; Figure 6E), and PK-45H (41% reduction; *p* < 0.01; Figure 6F) compared with control hIgG_1_. The resected CHO/GPC1, PC-10, and PK-45H tumors on day 20 are shown in each figure. The tumor-bearing mice did not lose body weight by G_1_Mab-28-hG_1_ treatment (Figure 6G–I).

## 3. Discussion

This study demonstrated the in vitro and in vivo antitumor efficacy of a novel mAb against GPC1. Both G_1_Mab-28-mG_2a_ and G_1_Mab-28-hG_1_ recognized CHO/GPC1, PC-10, and PK-45H in flow cytometry (Figure 2). In the same experimental setting, the ADCC, CDC (Figure 3 and Figure 5), and in vivo antitumor effect (Figure 4 and Figure 6) were observed in G_1_Mab-28-mG_2a_ and G_1_Mab-28-hG_1_. The binding affinity to CHO/GPC1 or PK-45H (Figure 1C and Figure 2D) and the in vitro/in vivo efficacy (Figure 3, Figure 4, Figure 5 and Figure 6) were similar between G_1_Mab-28-mG_2a_ and G_1_Mab-28-hG_1_, suggesting that G_1_Mab-28-hG_1_ activated the effectors and exerted antitumor efficacy in nude mice.

A chicken/mouse chimeric anti-GPC1 mAb (clone 1–12) exhibited the ADCC and CDC and inhibited the tumor growth of esophageal cancer patient-derived tumor inoculated in SCID or NOD/SCID mice [26]. Since the antitumor effect was observed in severe immunodeficient NOD/SCID mice and clone 1–12 was able to detect mouse GPC1 expressed in vascular endothelial cells in tumor microenvironments (TME), the antiangiogenic effect was also thought to be involved in the antitumor effect [26]. In contrast, G_1_Mab-28-mG_2a_ and G_1_Mab-28-hG_1_ did not recognize mouse GPC1 (Figure 1C). Therefore, both mAbs exerted the antitumor effect through the ADCC and CDC mainly. Furthermore, the reductions in tumor volume were observed at day 20 compared to that at day 7 (treatment start day, Figure 4 and Figure 6), suggesting that the monotherapy of G_1_Mab-28-mG_2a_ and G_1_Mab-28-hG_1_ is expected for tumor treatment. Additionally, the side effect to normal tissues cannot be expected in this study.

The above group next developed another anti-GPC1 mAb (clone 01a033) and the humanized version (clone T2), which has a high internalizing activity suitable for ADC [27,28,31]. In PDAC, GPC1 expression was elevated in both PDAC and cancer-associated fibroblasts (CAFs) in 80% of patients [39]. In a mouse xenograft model of PDAC patient-derived tumor with GPC1-positive CAF and tumor cells, the 01a033-ADC showed a potent antitumor effect [39]. These results indicate that targeting GPC1 on PDAC and CAF by the 01a033-ADC is a promising approach in stroma-rich PDAC. For the development of ADC in G_1_Mab-28-mG_2a_ and G_1_Mab-28-hG_1_, the epitope and internalizing activity should be investigated in future studies.

GPC1 expression in normal tissues has been considered minimal or absent. The distribution of GPC1 in normal tissues has primarily been evaluated by immunohistochemistry (IHC) [40,41]. However, our flow cytometric analyses demonstrated that G_1_Mab-28-mG_2a_ and G_1_Mab-28-hG_1_ recognize fibroblast, keratinocyte, and corneal epithelial cell lines (Figure 2D). As mentioned above, GPC1 was detected in TME, including tumor-infiltrating CAFs and/or vascular endothelial cells [26,39,42]. If anti-GPC1 mAbs act on normal epithelial or stromal cells, this is a concern to apply the modalities to clinical studies. For instance, ocular surface adverse events including dry eye, keratitis/keratopathy, blurred vision, conjunctivitis, and corneal pseudomicrocysts have been attributed to ADC treatment [43,44]. The ideal therapeutic targets are expected to be highly expressed in tumors but have no or minimal expression in normal tissues [45]. However, such tumor-associated antigens are limited in their use for the development of therapeutic mAbs.

To achieve a favorable therapeutic index while minimizing on-target toxicity, we have developed cancer-specific monoclonal antibodies (CasMabs) targeting antigens such as podocalyxin, podoplanin, and human epidermal growth factor receptor 2 (HER2) and have successfully identified the corresponding cancer-specific epitopes. An anti-HER2 CasMab, H_2_CasMab-2, was selected from approximately 300 anti-HER2 mAb clones [46]. H_2_CasMab-2 selectively recognized HER2 on breast cancer cells but showed no reactivity to normal epithelial cells derived from the mammary gland, kidney proximal tubule, lung bronchus, or colon in flow cytometry [46]. We also revealed the structural basis of the recognition between extracellular domain IV of HER2 and H_2_CasMab-2 [47]. Furthermore, a scFv derived from H_2_CasMab-2 was incorporated into CAR T cells, which demonstrated cancer-specific reactivity and significant antitumor efficacy in a preclinical study [47]. Currently, the H_2_CasMab-2 CAR-T therapy is under evaluation in a phase I clinical trial for patients with HER2-positive advanced solid tumors (NCT06241456). Collectively, these findings highlight the importance of selecting CasMabs against GPC1 and identifying their cancer-specific epitopes as key strategies for the development of therapeutic CasMabs and related modalities. We have established 124 clones of GPC1-targeting mAbs and will screen them for cancer-specific reactivity. Since GPC1 is broadly expressed in normal epithelial cells and fibroblastic cells, CasMab selection is important for minimizing the side effects. G_1_Mab-28-mG_2a_ and G_1_Mab-28-hG_1_ will be used as reference antibodies for the comparison of antitumor efficacy and toxicity to normal cells with anti-GPC1 CasMabs.

Accurate assessment of target expression is essential for determining eligibility for targeted therapies [48,49]. Evaluation of HER2 by IHC provides a semiquantitative measure of HER2 overexpression in the clinic [50]. Historically, limited attention has been paid to HER2-low tumors. However, the emergence of novel therapeutic agents that require fewer membrane epitopes for clinical efficacy has prompted a reassessment of current IHC protocols with particular emphasis on the lower limits of detection [51]. To facilitate the diagnosis of GPC1-positive tumors, standardization of the IHC protocol is essential. However, in several preclinical studies of anti-GPC1 therapies, polyclonal antibodies have been used to detect GPC1 in formalin-fixed paraffin-embedded (FFPE) tumor sections [27,29,30,31]. Therefore, an anti-GPC1 mAb suitable for IHC is required. G_1_Mab-28 can stain the CHO/GPC1 section using an automated IHC platform [33]. However, G_1_Mab-28 was not able to stain the FFPE sections of human tumors, suggesting that conformational changes by antigen retrieval and/or the inaccessibility of the mAb may prevent the detection of GPC1 by G_1_Mab-28. We have screened the clones that are suitable for IHC to detect GPC1 from the abovementioned G_1_Mab clones, which would contribute to the standardization and the development of companion diagnosis for GPC1-positive tumors.

## 4. Materials and Methods

### 4.1. Cell Lines

A human lung squamous cell carcinoma cell line PC-10 was purchased from Immuno-Biological Laboratories Co., Ltd. (Gunma, Japan). A human PDAC cell line PK-45H and an embryonic fibroblast cell line KMST-6 were obtained from the Cell Resource Center for Biomedical Research, Institute of Development, Aging and Cancer, Tohoku University (Miyagi, Japan). A human keratinocyte cell line HaCaT was obtained from Cell Lines Service GmbH (Eppelheim, Germany). A human corneal epithelial immortalized cell line hTCEpi was purchased from EVERCYTE (Vienna, Austria). KYSE770 was obtained from the Japanese Collection of Research Bioresources (Osaka, Japan). A human GPC1-overexpressed Chinese hamster ovary-K1 (CHO/GPC1) cell line was previously established [33]. These cell lines were cultured as described previously [33,52].

The mouse GPC1 (NM_016696.4) cDNA was obtained from OriGene Technologies, Inc. (Rockville, MD, USA). The mouse GPC1 cDNA was cloned into a pCAG-Ble-ssnPA16 vector. The plasmid was transfected into CHO-K1, and stable transfectants were established by sorting with an anti-PA16 mAb, NZ-1, using a cell sorter.

GPC1-knockout KYSE770 (BINDS-70) was generated using the CRISPR/Cas9 system. A GPC1-specific sgRNA targeting the sequence 5′- CGTTCAGCAGGTGCTGGAAG -3′ (TrueGuide™ Synthetic sgRNA, Thermo Fisher Scientific, Inc., Waltham, MA, USA) was synthesized and cloned into the GeneArt™ CRISPR Nuclease OFP Vector (Thermo Fisher Scientific, Inc.).

### 4.2. Antibodies

To generate recombinant mouse IgG_2a_-type G_1_Mab-28 (G_1_Mab-28-mG_2a_) and human IgG_1_-type G_1_Mab-28 (G_1_Mab-28-hG_1_), the V_H_ and V_L_ cDNAs of G_1_Mab-28 (mouse IgG_1_, κ) were cloned into pCAG-Neo and pCAG-Ble vectors together with the corresponding constant regions of mouse IgG_2a_ [53] and human IgG_1_ [54], respectively. The antibody expression vectors were transfected into ExpiCHO-S cells using the ExpiCHO Expression System to produce G_1_Mab-28-mG_2a_ and G_1_Mab-28-hG_1_. The PMab-231 (for isotype control mouse IgG_2a_) [53] and humCvMab-62 (for isotype control human IgG_1_) [54] were also prepared. All antibodies were purified using Ab-Capcher (ProteNova Co., Ltd., Kagawa, Japan). These mAbs were denatured by an SDS sample buffer (Nacalai Tesque, Inc., Kyoto, Japan) containing 2-mercaptoethanol and subjected to SDS-PAGE. The gel was stained with Bio-Safe CBB G-250 Stain (Bio-Rad Laboratories, Inc., Berkeley, CA, USA).

### 4.3. Animals

The animal study for the antitumor efficacy of G_1_Mab-28-mG_2a_ and G_1_Mab-28-hG_1_ was approved by the Institutional Committee for Experiments of the Institute of Microbial Chemistry (approval no. 2025-045), within which the work was undertaken, confirming that it conforms to the provisions of the Declaration of Helsinki. Humane objectives for euthanasia were established as a loss of original body weight to a point of >25% and/or a maximal tumor size of >3000 mm^3^.

### 4.4. Flow Cytometry and Determination of Binding Affinity

Cells were harvested using 1 mM ethylenediaminetetraacetic acid in phosphate-buffered saline (PBS). The cells were treated with primary mAbs in blocking buffer (0.1% bovine serum albumin in PBS) for 30 min at 4 °C. Then, the cells were treated with Alexa Fluor 488-conjugated anti-mouse or rat IgG (1:2000; Cell Signaling Technology, Inc., Danvers, MA, USA), or fluorescein isothiocyanate (FITC)-conjugated anti-human IgG (1:2000; Sigma-Aldrich Corp., St. Louis, MO, USA) for 30 min at 4 °C. Fluorescence data were collected using the SA3800 Cell Analyzer (Sony Corp., Tokyo, Japan) and analyzed with FlowJo software (version 10.8.1, BD Biosciences, Franklin Lakes, NJ, USA).

Cells were treated with serially diluted primary mAbs. Subsequently, the cells were incubated with Alexa Fluor 488-conjugated anti-mouse IgG (200-fold dilution) for 30 min at 4 °C. Data were collected using the SA3800 Cell Analyzer, and the geometric mean (GeoMean) was determined with FlowJo. The dissociation constant (*K*_D_) values were calculated using GraphPad PRISM 6 (version 6.07, GraphPad Software, Inc., La Jolla, CA, USA).

### 4.5. Antibody-Dependent Cellular Cytotoxicity

Five-week-old female BALB/c nude mice were purchased from Japan SLC, Inc. (Shizuoka, Japan). Effector cells were isolated from the spleens as described previously [55]. Target cells (CHO/GPC1, PC-10, and PK-45H) were labeled with 10 µg/mL of Calcein AM (Thermo). The target cells were plated in 96-well plates at a density of 5 × 10^3^ cells/well and combined with effector cells (effector-to-target ratio, 50:1) and 100 μg/mL of either control mIgG_2a_ or G_1_Mab-28-mG_2a_, or either control hIgG_1_ or G_1_Mab-28-hG_1_. After incubating for 4 h at 37 °C, the Calcein released into the supernatant was measured as described previously [56].

### 4.6. Complement-Dependent Cytotoxicity

The target cells labeled with Calcein AM (CHO/GPC1, PC-10, and PK-45H) were seeded and combined with rabbit complement (final concentration 10%, Low-Tox-M Rabbit Complement; Cedarlane Laboratories, Hornby, ON, Canada) along with 100 μg/mL of either control mIgG_2a_ or G_1_Mab-28-mG_2a_, or either control hIgG_1_ or G_1_Mab-28-hG_1_. After a 4 h incubation at 37 °C, the amount of Calcein released into the medium was measured as described previously [56].

### 4.7. Antitumor Activities in Xenografts of Human Tumors

CHO/GPC1, PC-10, and PK-45H were mixed with Matrigel Matrix Growth Factor Reduced (BD Biosciences). Subcutaneous injections (5 × 10^6^ cells/mouse) were then given to the left flanks of BALB/c nude mice. On the seventh post-inoculation day, 100 µg of control mIgG_2a_ (n = 8), G_1_Mab-28-mG_2a_ (n = 8), control hIgG_1_ (n = 8), or G_1_Mab-28-hG_1_ (n = 8) in 100 µL PBS were administered intraperitoneally. Additional antibody injections were given on day 13. The tumor diameter was assessed on days 7, 10, 13, 17, and 20 after the tumor cell implantation. Tumor volume was calculated using the formula: volume = W^2^ × L/2, where W represents the short diameter and L the long diameter. The mice’s weight was also assessed on days 7, 10, 13, 17, and 20 following tumor cell inoculation. When observations on day 20 were complete, the mice were sacrificed, and tumor weights were assessed after tumor excision.

### 4.8. Statistical Analyses

The mean ± standard error of the mean (SEM) is presented in all data. A two-tailed unpaired *t*-test was conducted to measure ADCC, CDC, and tumor weight. ANOVA with Sidak’s post hoc test was performed for tumor volume and mouse weight. GraphPad Prism 10 (version 10.6.1, GraphPad Software, Inc.) was used for all calculations. *p* < 0.05 was considered statistically significant.

## Data Availability

The data presented in this study are available in the article.

## Supplementary Materials

The following supporting information can be downloaded at: https://www.mdpi.com/article/10.3390/ijms27104181/s1.
