# Supplementary material for: Chimeric Anti-Glypican 1 Antibodies Exert Antitumor Activities in Xenograft Models of Lung and Pancreatic Cancers
Source: Int J Mol Sci. 2026 May 8;27(10):4181. doi: 10.3390/ijms27104181 (PMC13207206; doi:10.3390/ijms27104181)
Supplement: Supplementary file 1 [file ijms-27-04181-s001.zip › ijms-4180204-supplementary.pdf]

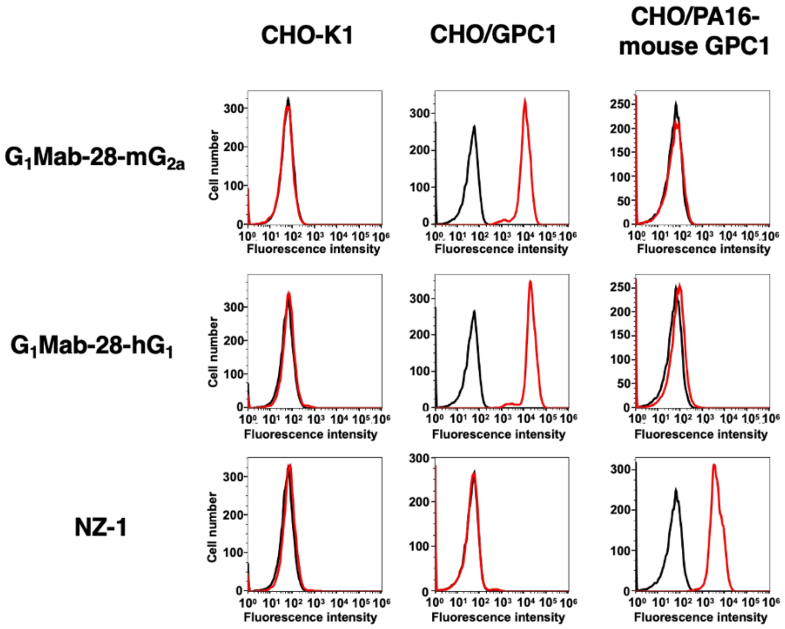

Supplementary figure S1. Reactivity of G_1Mab-28-mG_{2a} and G_1Mab-28-hG_1 to human and mouse GPC1. CHO/GPC1, CHO/PA16-mouse GPC1, and CHO-K1 were treated with G_1Mab-28-mG_{2a} (1 µg/mL; Red line), G_1Mab-28-hG_1 (1 µg/mL; Red line), an anti-PA16 tag mAb (NZ-1, 1 µg/mL; Red line), or buffer control (Black line). After treatment with primary mAbs, cells were treated with Alexa Fluor 488-conjugated anti-mouse or rat IgG, or FITC-conjugated anti-human IgG. Fluorescence data were analysed using the SA3800 Cell Analyzer. Merged histograms of CHO/GPC1 or CHO/PA16-mouse GPC1 were presented in Fig. 1C.

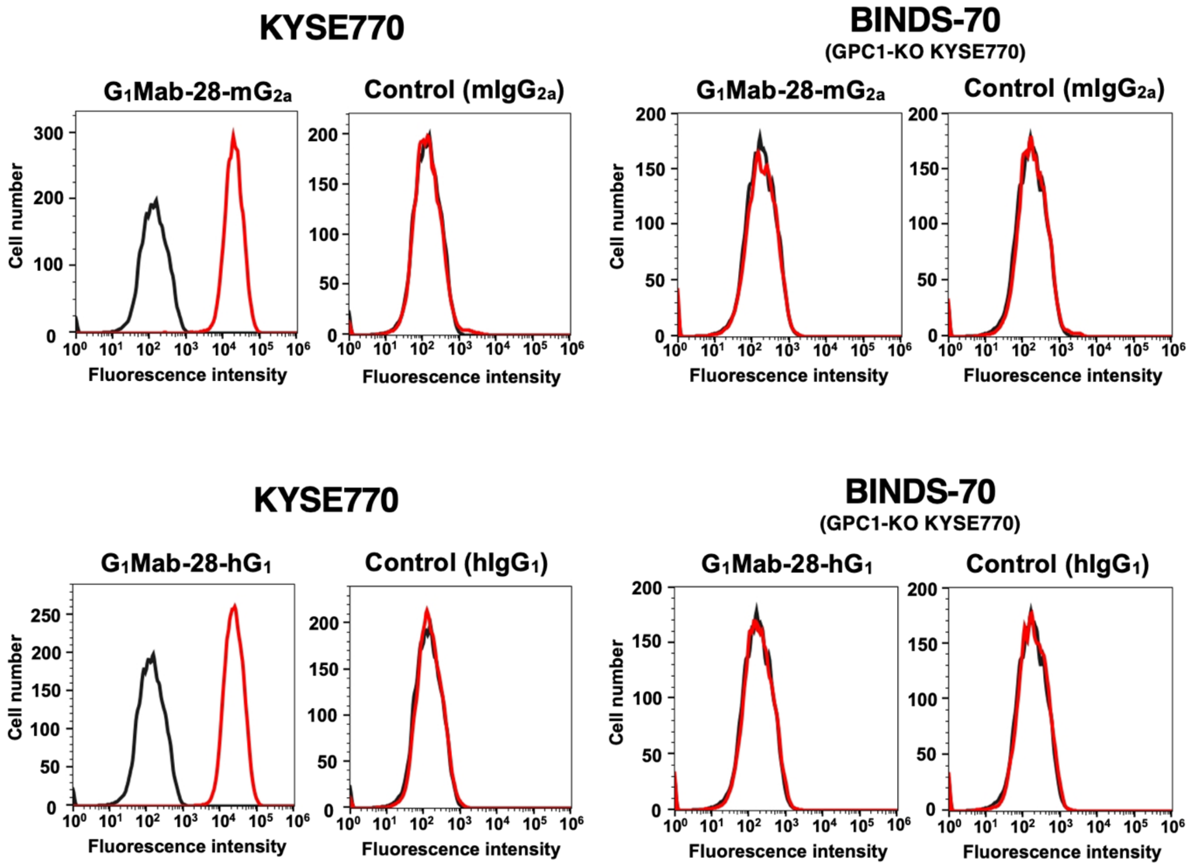

Supplementary figure S2. Flow cytometric analysis using GPC1-knockout (KO)-KYSE770. KYSE770 and BINDS-70 (GPC1-KO KYSE770) were treated with 1 μg/mL of an isotype control IgG_{2a} mAb (PMab-231, red) or 1 μg/mL of G_1Mab-28-mG_{2a} (red). The mAbs-treated cells were incubated with anti-mouse IgG conjugated with Alexa Fluor 488. These cells were treated with 1 μg/mL of an isotype control human IgG_1 mAb (humCvMab-62, red) or 1 μg/mL of G_1Mab-28-hG_1 (red). The mAbs-treated cells were incubated with anti-human IgG conjugated with FITC. The fluorescence data were collected using the SA3800 Cell Analyzer.

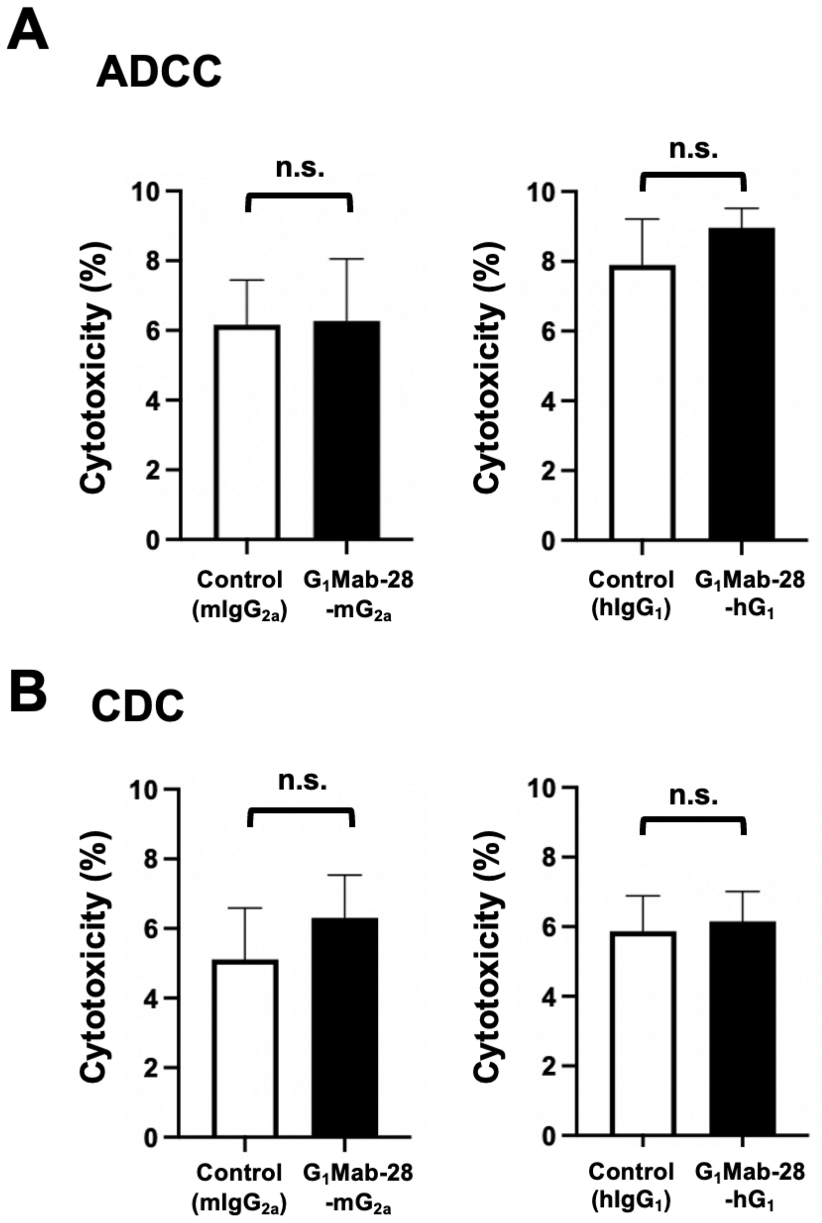

Supplementary figure S3. ADCC (A) and CDC (B) assays were performed using GPC1-KO KYSE770 (BINDS-70). Calcein release into the medium was measured, and cytotoxicity was determined. Values are shown as the mean ± SEM (n = 3). Asterisks indicate statistical significance (* $p < 0.05$; two-tailed unpaired t-test). n.s., not significant.
